# Elucidating the mitochondrial function of murine lymphocyte subsets and the heterogeneity of the mitophagy pathway inherited from hematopoietic stem cells

**DOI:** 10.3389/fimmu.2022.1061448

**Published:** 2022-11-07

**Authors:** Haoyue Liang, Weichao Fu, Wenying Yu, Zhijie Cao, Ertao Liu, Fanfan Sun, Xiaodong Kong, Yingdai Gao, Yuan Zhou

**Affiliations:** ^1^ State Key Laboratory of Experimental Hematology, National Clinical Research Center for Blood Diseases, Haihe Laboratory of Cell Ecosystem, Institute of Hematology & Blood Diseases Hospital, Chinese Academy of Medical Sciences & Peking Union Medical College, Tianjin, China; ^2^ Department of Geriatrics, Tianjin Geriatrics Institute, Tianjin Medical University General Hospital, Tianjin, China

**Keywords:** metabolic map, mitophagy, hematopoietic stem cell, mitochondrial functions, lymphocyte subsets

## Abstract

**Background:**

Mitochondria are mainly involved in ATP production to meet the energy demands of cells. Researchers are increasingly recognizing the important role of mitochondria in the differentiation and activation of hematopoietic cells, but research on how mitochondrial metabolism influence different subsets of lymphocyte at different stages of differentiation and activation are yet to be carried out. In this work, the mitochondrial functions of lymphocytes were compared at different differentiation and activation stages and included CD8^+^ T lymphocytes, CD4^+^ T lymphocytes, B lymphocytes, NK cells as well as their subsets. For this purpose, a complete set of methods was used to comprehensively analyze mitophagy levels, mitochondrial reactive oxygen species (ROS), mitochondrial membrane potential (MMP) and the mitochondrial mass (MM) of subsets of lymphocytes. It is expected that this will provide a complete set of standards, and drawing the mitochondrial metabolic map of lymphocyte subsets at different stages of differentiation and activation.

**Results and discussion:**

Of all lymphocytes, B cells had a relatively high mitochondrial metabolic activity which was evident from the higher levels of mitophagy, ROS, MMP and MM, and this reflected the highly heterogeneous nature of the mitochondrial metabolism in lymphocytes. Among the B cell subsets, pro-B cells had relatively higher levels of MM and MMP, while the mitochondrial metabolism level of mature B cells was relatively low. Similarly, among the subsets of CD4^+^ T cell, a relatively higher level of mitochondrial metabolism was noted for naive CD4^+^ T cells. Finally, from the CD8^+^ T cell subsets, CD8^+^ Tcm had relatively high levels of MM and MMP but relatively low ones for mitophagy, with effector T cells displaying the opposite characteristics. Meanwhile, the autophagy-related genes of lymphoid hematopoietic cells including hematopoietic stem cells, hematopoietic progenitor cells and lymphocyte subsets were analyzed, which preliminarily showed that these cells were heterogeneous in the selection of mitophagy related Pink1/Park2, BNIP3/NIX and FUNDC1 pathways. The results showed that compared with CD4^+^ T, CD8^+^ T and NK cells, B cells were more similar to long-term hematopoietic stem cell (LT-HSC) and short-term hematopoietic stem cell (ST-HSC) in terms of their participation in the Pink1/Park2 pathway, as well as the degree to which the characteristics of autophagy pathway were inherited from HSC. Compared with CLP and B cells, HSC are less involved in BNIP3/NIX pathway. Among the B cell subsets, pro-B cells inherited the least characteristics of HSC in participating in Pink1/Park2 pathway compared with pre-B, immature B and immature B cells. Among CD4^+^ T cell subsets, nT_reg_ cells inherited the least characteristics of HSC in participating in Pink1/Park2 pathway compared with naive CD4^+^ T and memory CD4^+^ T cells. Among the CD8^+^ T cell subsets, compared with CLP and effector CD8^+^ T cells, CD8^+^ Tcm inherit the least characteristics of HSC in participating in Pink1/Park2 pathway. Meanwhile, CLP, naive CD4^+^ T and effector CD8^+^ T were more involved in BNIP3/NIX pathway than other lymphoid hematopoietic cells.

**Conclusion:**

This study is expected to provide a complete set of methods and basic reference values for future studies on the mitochondrial functions of lymphocyte subsets at different stages of differentiation and activation in physiological state, and also provides a standard and reference for the study of infection and immunity based on mitochondrial metabolism.

## Introduction

The immune system includes the innate and adaptive immunity which can protect the body against damage caused by microorganisms from the surrounding environment (1, 2). It involves interactions between different types of different cells, with lymphocytes and their subsets forming the basis of this defense mechanism. Basically, lymphocytes include NK cells, T cells and B cells which, after proliferation and differentiation from hematopoietic stem cells, go on to produce their corresponding precursor cells that eventually differentiate into mature cells. For example, hematopoietic stem cells first divide asymmetrically to produce lymphoid progenitor cells which are common to both B and T cells. The former then differentiate from pro-B cells into pre-B cells that subsequently undergo additional differentiation to produce immature and finally mature B cells. On the other hand, T cells need to go through positive and negative selection processes to first yield CD8^+^ and CD4^+^ T cells prior to their subsequent differentiation into various T cells with specific functions. In this case, the subset of CD8^+^ T cell include effector CD8^+^ T, effector memory CD8^+^ (CD8^+^ Tem) and central memory CD8^+^ T (CD8^+^ Tcm), while the subset for CD4^+^ T cells include nT_reg_ cells, memory CD4^+^ and naive CD4^+^ T cells (3, 4).

Mitochondria, one of the most important organelles in cells, is involved in producing ATP, regulating the formation of reactive oxygen species, maintaining calcium balance as well as participating in signal transmission, inflammation and cell death (5, 6). Changes in mitochondrial functions due to oxidative phosphorylation injuries, autophagy disorders, inhibition of apoptosis, abnormal energy metabolism, changes in signal pathways and promotion of immune escape, may trigger the occurrence of diseases, with some common examples being blood system diseases, autoimmune diseases, cardiovascular and cerebrovascular diseases, diabetes and malignant tumors which are closely related to dysfunctions in mitochondrial activities (7–9). In addition to mitochondrial functions, the organelle’s characteristics such as mitophagy and reactive oxygen species (ROS) levels, mitochondrial membrane potential (MMP) and mitochondrial mass (MM) also play important roles in the differentiation and activation of different subsets of lymphocyte. These features reflect the role of mitochondria in physiological and pathological processes such as cell activity, stress and aging.

When immune responses are triggered, immune cells are activated and they change from a relatively static metabolic state to a highly active one (10). At this stage, the cells’ metabolism needs to not only meet the requirements for ATP and metabolic intermediates but also to act as a feedback mechanism that provide signals to drive the required phenotypic changes of immune cells (11). Therefore, the metabolism of immune cells is very important for the cells’ functions and at the center of this process are the mitochondria which contribute to the differentiation and maturation of immune cells. In this context, researchers generally believe that the heterogeneity of mitochondrial functions causes B cells to undergo a significant change from an initially quiescent state to anabolically-active proliferating cells. During the subsequent clonal expansion of B cells, the metabolism of glucose and glutamine is then significantly enhanced (12), with other mitochondria-derived compounds also contributing to B cell activation as well as its effector functions. Furthermore, it has been reported that, compared with primitive B cells, the level of phospholipids produced by plasma cells also increases, with plasma cell differentiation depending on the conversion of citrate from the mitochondria to cytosolic acetyl CoA and oxaloacetic acid (13). The above suggests that mitochondrial processes are important for B cell activation as well as for the development of plasma cells.

Similarly, the heterogeneity of mitochondrial functions also produces various subsets of inflammatory and inhibitory CD4^+^ T helper cells, with differences in the role of mitochondrial metabolism being particularly obvious when comparing T_reg_ cells and Th17 cells. Indeed, compared with Th17 cells, the level of oxidative phosphorylation of T_reg_ cells is higher but glycolysis levels are lower (14). At the same time, mitochondrial metabolism can regulate the formation of T cells. For example, T cell activation results in the rapid uptake of glucose and glutamine for producing ATP as well as the other cellular component needed for cloning effector T cells. In addition, the mitochondria of effector T cells regulate anabolic processes, while for memory T cells, ATP is produced through the decomposition and metabolism of fatty acids to promote cell survival (11). As far as NK cells are concerned, cellular development and other processes, including proliferation is dependent on glycolysis and oxidative phosphorylation (15). In fact, for quiescent mature NK cells, oxidative phosphorylation is generally sufficient to effectively generate the energy required for cell balance, without the need to invest additional energy in the synthesis of biochemical molecules. However, on activation, this metabolic state is changed. However, the above studies lack a complete comparison of mitochondrial metabolic functions between lymphocytes at different stages of differentiation and activation.

Since an immune cell’s metabolism is not only a means of producing energy and other biomolecules but also determines the cell’s immune output, an in-depth study of the mitochondrial metabolic pathway of immune cells can provide a new strategy for the treatment of cancer and other immune-related dysfunctions. For this purpose, this work aimed to detect the ROS, MMP, MM and mitochondrial autophagy levels of lymphocytes at different stages of differentiation and activation. By using similar experimental conditions and system standards, the characteristics of mitochondrial metabolism of these lymphocytes were explored in view of providing a set of reference methods and values for future studies on the immune-related functions of mitochondrial metabolism. Furthermore, an analysis of the heterogeneity of the mitochondrial autophagy pathway in these lymphocytes was performed based on single cell sequencing. It is expected that these additional results will provide a foundation for future studies that examine how mitochondrial functions could be involved in malignant tumors, aging as well as blood, cardiovascular and cerebrovascular diseases.

## Materials and methods

### Mice

C57BL/6J (B6) mice were maintained in the animal facility of the Institute of Hematology. All experiments involving animals were carried out according to the animal care guidelines with approval of the Institutional Animal Care and Use Committees of the State Key Laboratory of Experimental Hematology.

Fourteen subpopulations of lymphocytes consisting of CD8^+^ T, CD4^+^ T, B and Natural Killer cells (NK) as well as their subsets (effector CD8^+^ T, CD8^+^ Tem, CD8^+^ Tcm, naive CD8^+^ T, memory CD4^+^ T, naive CD4^+^ T, nT_reg_, pro-B, pre-B, immature B and mature B) were obtained from the flushed bone marrow of 6-8-week-old mice (**Figure S1**). The cells were stained and enriched as previously described (**Figure S2**) prior to flow cytometry on BD Aria™ flow cytometer (BD Biosciences, USA), with the results subsequently analyzed using FlowJo™ V10.6.1 (BD Biosciences, USA). **Table S1** show the cell surface markers used for immunophenotyping while **Tables S2–S6** show the antibodies used for staining.

### Mitochondrial function detection

**Figure 1:**
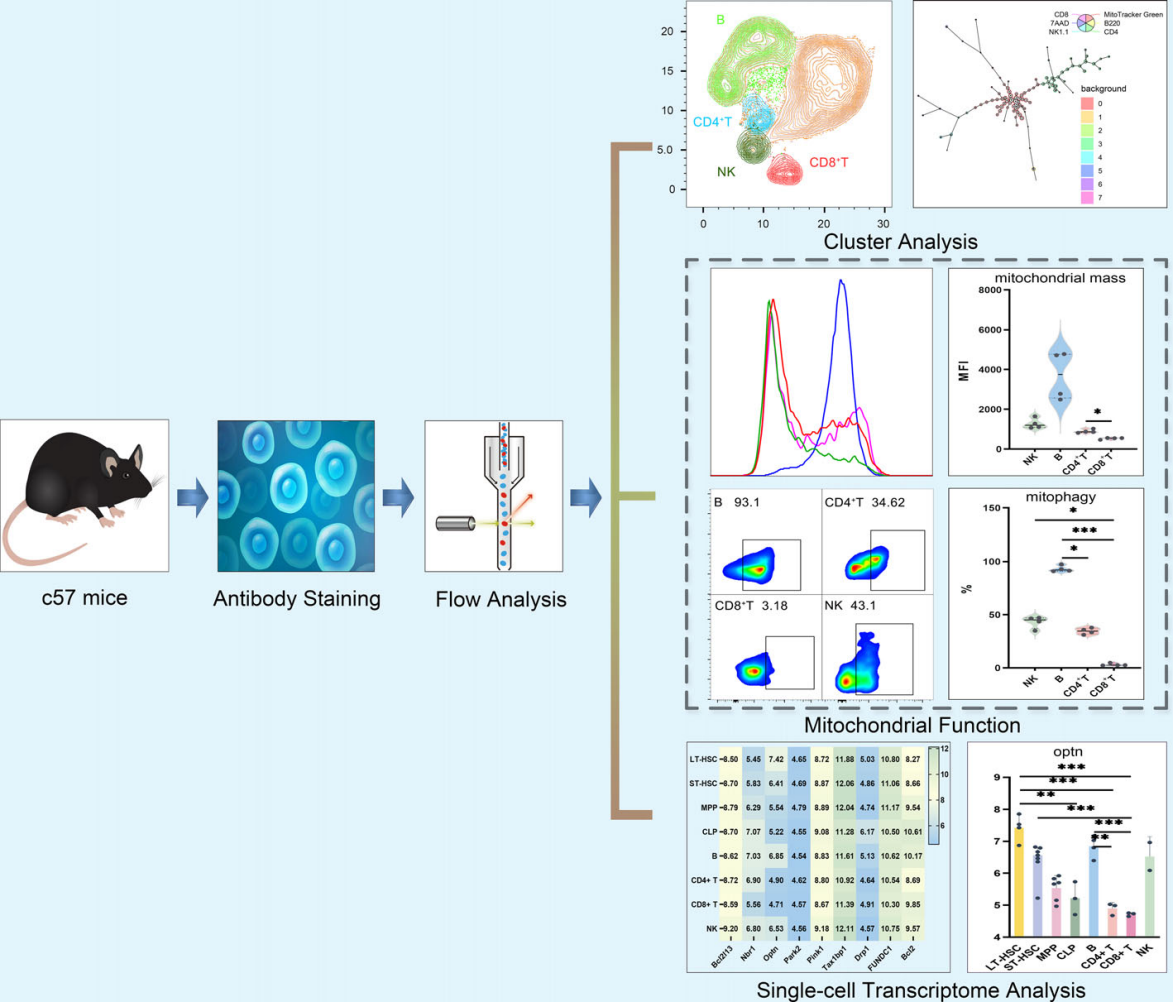
Schematic diagram of mitochondrial functions in different subsets of lymphocytes.

After isolating lymphocytes, the levels of mitophagy, mitochondrial ROS, MMP and MM were measured based on flow cytometry (BD Biosciences, USA). For this purpose, cell concentrations were adjusted to 5×10^6^ cells/ml for each tube and after adding combinations of antibodies as listed in **Tables S2–S6**, incubation was performed for 30 min at 4°C. This was followed by the addition of 3 ml of phosphate buffer saline (PBS) prior to centrifugation for 5 min at 1500 rpm. The supernatant was then discarded and after adding 500 μl of a working solution for detecting mitochondrial function, incubation was performed in the dark for 20 min at 37°C. The addition of PBS (3 ml) and subsequent centrifugation was then repeated as before. with the resulting pellet washed twice using a similar procedure. The pellet was dissolved in 500 μl PBS and after filtering the solution using a 30–70 μm nylon mesh, 3 μl of 7-AAD staining solution was added, with the results analyzed within 1 h. In this case, mitophagy, ROS, MMP and MM were detected by using Mitophagy Dye working solution (100 nM), MitoSOX Red working solution (5 μM), Mitotracker Red working solution (30 nM) and Mitotracker Green working solution (100 nM) respectively. The detailed procedure is provided as supplementary information while **Figure 1** shows a schematic diagram of the study of mitochondria in different subsets of lymphocytes.

### Analysis of single-cell transcriptome data

Single-cell transcriptome data for hematopoietic stem cells (HSCs), multipotent progenitor cells (MPPs), common lymphoid progenitor cells (CLPs), and lymphocytes of mouse bone marrow were extracted from the NCBI database (GSE77098). This was followed by an analysis of nine genes related to mitophagy in blood cells to determine their expression levels.

### Data analysis

For all flow cytometry data, the mean fluorescence intensity and standard deviation (SD) of each channel were determined. For the t-distributed stochastic neighbor embedding (t-SNE) and uniform manifold approximation and projection (UMAP) diagrams, cells for which protein expression was similar are close, while highly interconnected nodes cells represent those having similar phenotypes in order to visualize different subsets of cells. A heat map or a color dimension overlaid on the t-SNE or UMAP diagrams show the final cell classification. In the t-SNE and UMAP diagrams, the proximity of cells reflects their distance in the high-dimensional space.

Data processing was carried out with the SPSS 26.0 statistical software package. Normally-distributed data were presented as the mean ± SD, with the results compared using ANOVA. Groups with a uniform variance or an uneven one were compared by applying the LSD method or Tamhane’s T2 method respectively. For non-normally distributed data, the results were expressed as median with the interquartile range. In addition, with the distribution being non-normal in this case, groups were compared using the non-parametric Kruskal-Wallis test. For all statistical tests, significant differences were indicated by *P-values* of < 0.05. The GraphPad Prism 6 software were used for all graphical representations.

## Results

### Mitochondrial functions of lymphocytes

**Figure 2:**
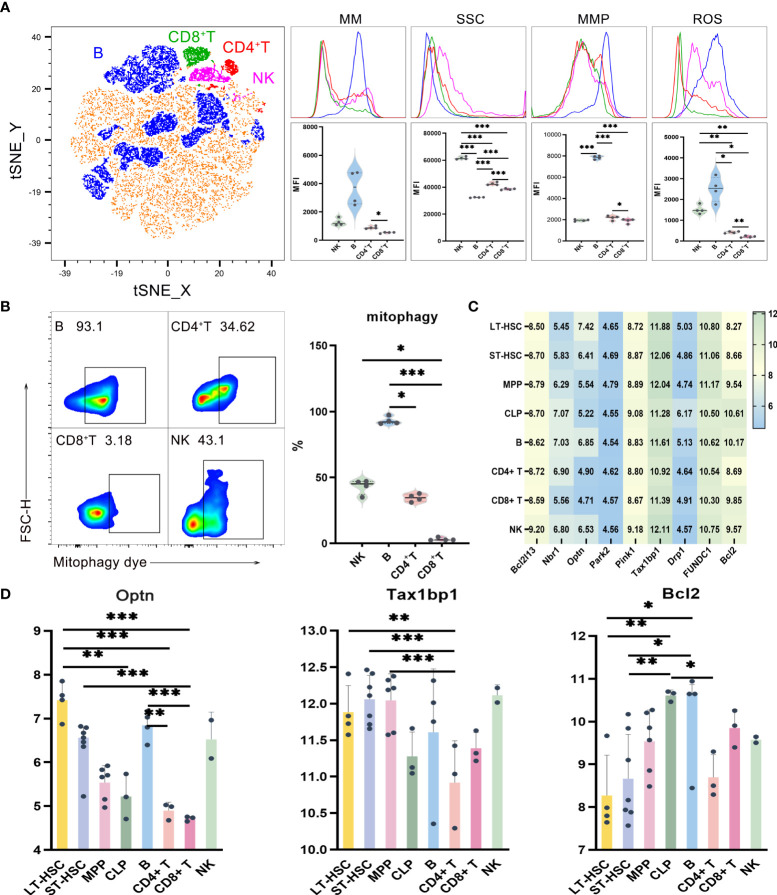
**(A)** t-SNE, MM, SSC, MMP and ROS levels in B, CD4^+^ T, CD8^+^ T and NK cells **(B)** Mitophagy in B, CD4^+^ T, CD8^+^ T and NK cells **(C)** Heat map for the expression level of autophagy-related genes in different types of hematopoietic cells during lymphoid differentiation **(D)** Statistics for the expression level of autophagy-related genes. Normally-distributed data are presented as the mean ± SD, while non-normally-distributed ones are presented as the median with the interquartile range. One-way ANOVA or the Kruskal–Wallis test was used for comparisons and determining the *P* values. *P<0.05, **P<0.01, ***P<0.001.

UMAP, t-SNE and FlowSOM analyses showed that lymphocytes could be divided into subgroups based on their different phenotypes (**Figure 2A**, **Figure S3**). In order to study the mitochondrial functions of these different subgroups, the levels of mitophagy, ROS, MMP and MM were then analyzed, with the results, reflecting the mitochondrial functions of T, B and NK cells, shown in **Figures 2A, B**. Globally, out of the four lymphocytes, the level of mitochondrial function was the highest for B cells, followed by NK and CD4^+^ T cells, with the lowest level obtained for CD8^+^ T. More specifically, compared with CD8^+^ T cells, CD4^+^ T cells had significantly higher levels of MM, MMP and ROS. Similarly, B cells had the highest level of MMP (*P* < 0.001) as well as a significantly higher mitophagy level compared with CD4^+^ and CD8^+^ T cells, with NK cells also having a significantly higher mitophagy level than CD8^+^ T cells (**Figure 2B**). Finally, as far as ROS were concerned, the levels were significantly higher for both B and NK cells compared with CD4^+^ T and CD8^+^ T cells (**Figure 2A**).

In order to understand the expression level of autophagy-related genes in mouse lymphocytes, the results of flow cytometry were combined with those obtained after the analysis of data on single-cell gene expression extracted from the NCBI database. The flow sorting phenotype of lymphocytes, as observed from the database, was consistent with the one which was experimentally determined (**Figure S2A**). The expression of genes related to autophagy in different types of cells within the lymphoid lineage is shown in the heat map and this lays the foundation for exploring the heterogeneity of mitochondrial pathway of the lymphocytes inheriting hematopoietic stem cells (**Figure 2C**). The main autophagy-related genes which were studied include *Tax1bp1*, *Pink1*, *Park2*, *Drp1*, *FUNDC1*, *Bcl-2*, *Optn*, *Nbr1* and *Bcl2l13*, with the results indicating that their expression levels were significantly different in different populations (**Figure 2D**). Of these, *Bcl-2* is mostly involved in the BNIP3/NIX pathway while *Drp1* and *FUNDC1* mostly take part in the FUNDC1 pathway. As for the remaining genes, they are mostly involved in the Pink1/Park2 pathway These results indicate that those three pathways could be common during the process of mitochondrial autophagy in different hematopoietic stem progenitor cells and lymphocytes, and it is likely that they could be involved in the recruitment of autophagy-related proteins. Furthermore, the results shows that the heterogeneity of mitophagy in HSCs, MPPs, CLPs and lymphocytes during lymphoid differentiation is reflected not only in the number of mitophagy, but also in the heterogeneity of dominant pathways of mitophagy in different cells.

Statistical analysis of the expression levels of autophagy-related genes in lymphoid cells at different stages of differentiation showed that *Optn* was significantly more expressed in LT-HSC compared with CLP, CD8^+^ T and CD4^+^ T cells (*P* < 0.01). Similarly, a significantly higher expression level was noted for ST-HSC compared with CD8^+^ T cells (*P* < 0.001), while in the case of B, *Optn* expression was significantly higher than for CD4^+^ T and CD8^+^ T cells (*P* < 0.01). The other gene, *Tax1bp1*, encodes an autophagy receptor that connects ubiquitin to the membrane of an autophagosome membrane during the selective autophagic clearance of damaged mitochondria. In this case, a significantly lower expression level of this gene was noted for CD4^+^ T compared with LT-HSC, ST-HSC and MPP (*P* < 0.01). Altogether, the results suggested that out of the four lymphocytes, B cells were more similar to LT-HSC and ST-HSC in their level of involvement in the Pink1/Park2 pathway as well as in terms of their greater similarity to the autophagy pathway characteristics of HSC. In contrast, CD4^+^ T, CD8^+^ T and NK cells were less involved in the autophagy pathway and inherited less of the characteristics of HSC. For the BNIP3/NIX pathway, the expression level of *Bcl2* was significantly lower in LT-HSC and ST-HSC compared with CLP and B cells (*P* < 0.05), thus indicating a lower involvement of HSC in this particular pathway. The bioinformatics and statistical analyses of these results showed that transcription was heterogeneous and subgroup-specific in hematopoietic stem progenitor cells and lymphocytes, with the expression of autophagy genes, in particular, being significantly heterogeneous among cell populations.

Side scatter characteristics (SSC) was used in order to characterize the number of organelles in lymphocytes as an indicator of the MM of hematopoietic cells. SSC reflects cells’ granularity, that is, the complexity of cell organelles in terms of their types and numbers and the SSC results of lymphocytes and their different subsets are shown in **Figure 2A**. Globally, it was noted that the out of the four cell types, NK cells had the highest SSC levels, followed by CD4^+^ T and CD8^+^ T cells, with B cells having the lowest level (*P* < 0.001). In lymphocytes, the fact that B cells had higher levels of mitochondrial functions but lower SSC levels indicate a significantly lower number of organelles such as ribosome, Golgi apparatus, endoplasmic reticulum and lysosome in B cells.

### Mitochondrial functions in the differentiation of B lymphocyte subsets

**Figure 3:**
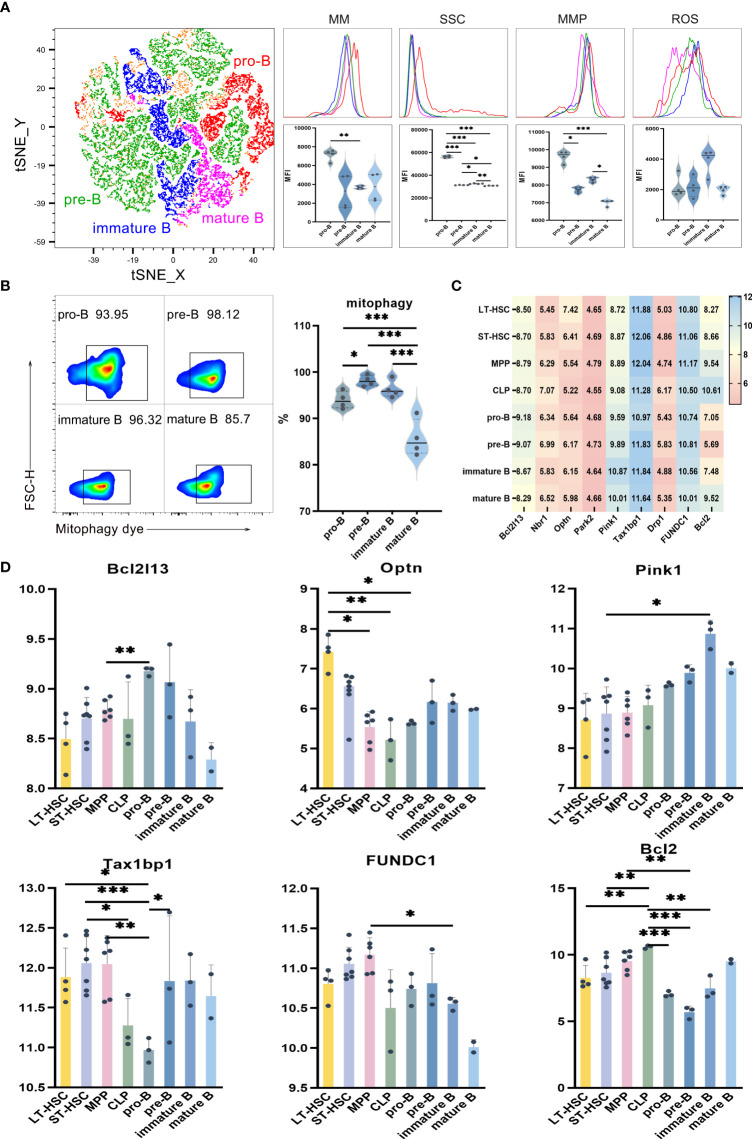
**(A)** t-SNE, MM, SSC, MMP and ROS levels in pro-B, pre-B, immature B and mature B cells **(B)** Mitophagy in pro-B, pre-B, immature B and mature B cells **(C)** Heat map for the expression level of autophagy-related genes in B cells at different differentiation stages **(D)** Statistics for the expression level of autophagy-related genes. Normally-distributed data are presented as the mean ± SD, while non-normally-distributed ones are presented as the median with the interquartile range. One-way ANOVA or the Kruskal–Wallis test was used for comparisons and determining the *P* values. *P<0.05, **P<0.01, ***P<0.001.

UMAP, t-SNE and FlowSOM analyses showed that, based on phenotypic differences, B lymphocytes could be subdivided into subsets. As previously described, the levels of mitophagy, ROS, MMP and MM were used in order to study the mitochondrial functions of these subsets, with the results for pro-B, pre-B, immature B and mature B cells shown in **Figures 3A, B**. Comparing B cells of different differentiation stages indicate significantly higher MM and MMP levels in pro-B compared with pre-B. Similar results were obtained when comparing the MM levels of pro-B with those of immature B, as well as when comparing immature B with mature B. These results suggest that more primitive subpopulations harbor higher MM levels than relatively more differentiated ones (**Figure 3A**). As far as SSC levels were concerned, they were significantly higher in poorly differentiated pro-B cells in comparison with mature B, immature B and pre-B cells (*P* < 0.001), thus indicating that the organelle content was higher in pro-B. Finally, results of mitophagy showed that, of all B cell subsets, pre-B had the highest mitophagy level while mature B had the lowest one. These results show that, compared with the relatively primitive subpopulation, mitophagy levels were lower in differentiated B cells (**Figure 3B**).

Statistical analyses of the expression of autophagy-related genes in B cells of different differentiation stages showed that *Optn* was significantly more expressed in LT-HSC compared with MPP, CLP and pro-B cells (*P* < 0.05). As a serine/threonine kinase, Pink1 can specifically locate depolarized mitochondria for activating parkin through ubiquitin phosphorylation. The recruitment of autophagy receptors subsequently induces mitophagy. *Pink1* expression was significantly lower in ST-HSC compared with immature B cells (*P* < 0.05), while that of *Tax1bp1* was significantly lower in pro-B in comparison with LT-HSC, ST-HSC, MPP and pre-B (*P* < 0.01). Thus, it appeared that, among the subsets of B cells, pro-B cells inherited the least characteristics of HSC in terms of their involvement in the Pink1/Park2 pathway. For the BNIP3/NIX pathway, *Bcl2* was significantly more expressed in CLP compared with LT-HSC, ST-HSC, pro-B, pre-B and immature B cells (*P* < 0.01), hence indicating that CLP was strongly involved in this pathway (**Figures 3C, D**).

### Mitochondrial functions in the activation of CD4^+^ T lymphocyte subsets

**Figure 4:**
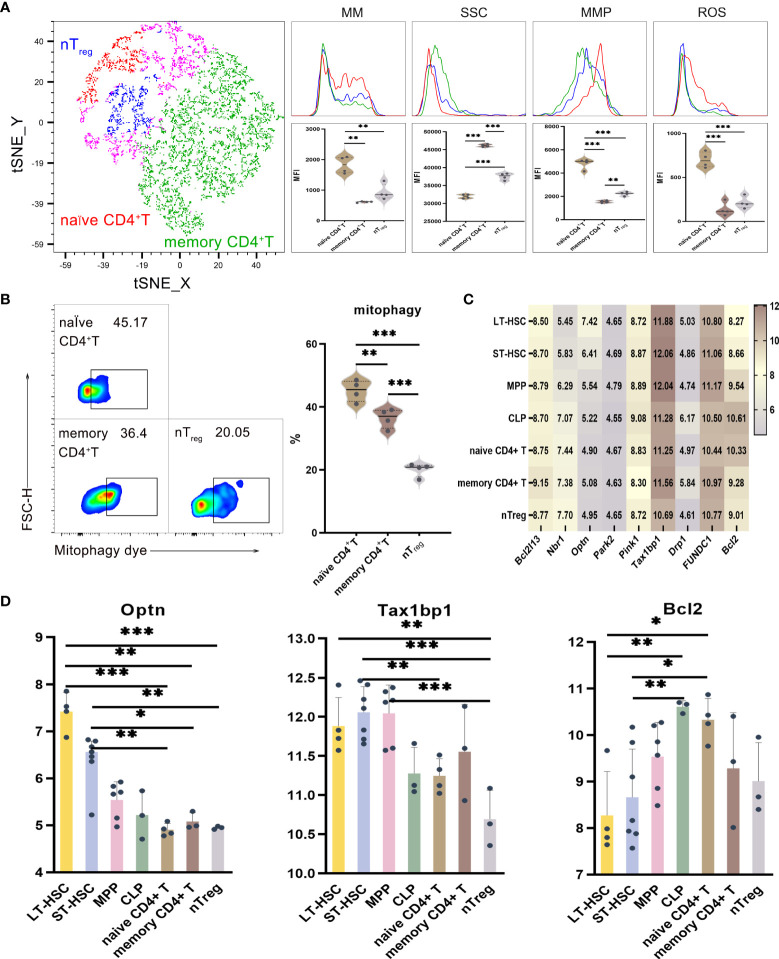
**(A)** t-SNE, MM, SSC, MMP and ROS in nT_reg_, memory CD4^+^ T and naive CD4^+^ T cells **(B)** Mitophagy in memory CD4^+^ T, naive CD4^+^ T and nT_reg_ cells **(C)** Heat map for the expression levels of autophagy-related genes in CD4^+^ T cell at different differentiation and activation stages **(D)** Statistics for the expression level of autophagy-related genes. Normally-distributed data are presented as the mean ± SD, while non-normally-distributed ones are presented as the median with the interquartile range. One-way ANOVA or the Kruskal–Wallis test was used for comparisons and determining the *P* values. *P<0.05, **P<0.01, ***P<0.001.

UMAP, t-SNE and FlowSOM analyses indicated that phenotypic differences allowed CD4^+^ T lymphocytes to be subdivided into different subsets. As described before, the levels of mitophagy, ROS, MMP and MM were analyzed to determine the mitochondrial functions of these CD4^+^ T cell subsets, with the results for memory CD4^+^ T, naive CD4^+^ T and nT_reg_ cells shown in **Figures 4A, B**. When comparing CD4^+^ T cells at different stages of differentiation, it was found that the MM, MMP, ROS and mitophagy levels were significantly higher for naive CD4^+^ T compared with memory CD4^+^ T and nT_reg_. Similarly, nT_reg_ had a significantly higher MMP level but a significantly lower mitophagy level than memory CD4^+^ T. Hence, naive CD4^+^ T subsets had higher mitophagy, ROS, MMP and MM levels in comparison with relatively more differentiated ones. As far as SSC levels were concerned, of all the subsets of CD4^+^ T, memory CD4^+^ T cells had the highest levels, followed by naive CD4^+^ T and with nT_reg_ cells having the lowest ones (*P* < 0.001). Therefore, the results reflected the heterogeneity in terms of organelle numbers.

Statistical analyses of the expression of autophagy-related genes for CD4^+^ T cell at different differentiation and activation stages indicated that *Optn* was more highly expressed in LT-HSC and ST-HSC compared with nT_reg_, memory CD4^+^ T and naive CD4^+^ T cells (*P* < 0.05). In addition, nT_reg_ had a significantly lower *Tax1bp1* expression level than LT-HSC, ST-HSC and MPP (*P* < 0.01), although that of ST-HSC was significantly higher compared with naive CD4^+^ T cells (*P* < 0.01). These results suggest that, of all subsets of CD4^+^ T cells, nT_reg_ cells inherited the least characteristics of HSC in terms of their participation in the Pink1/Park2 pathway. Regarding the BNIP3/NIX pathway, *Bcl2* expression was significantly higher in naive CD4^+^ T cells in comparison with LT-HSC and ST-HSC cells (*P* < 0.01), thus showing that in a similar way to CLP, the former was more involved in the BNIP3/NIX pathway (**Figures 4C, D**).

### Mitochondrial functions in the activation of CD8^+^ T lymphocyte subsets

**Figure 5:**
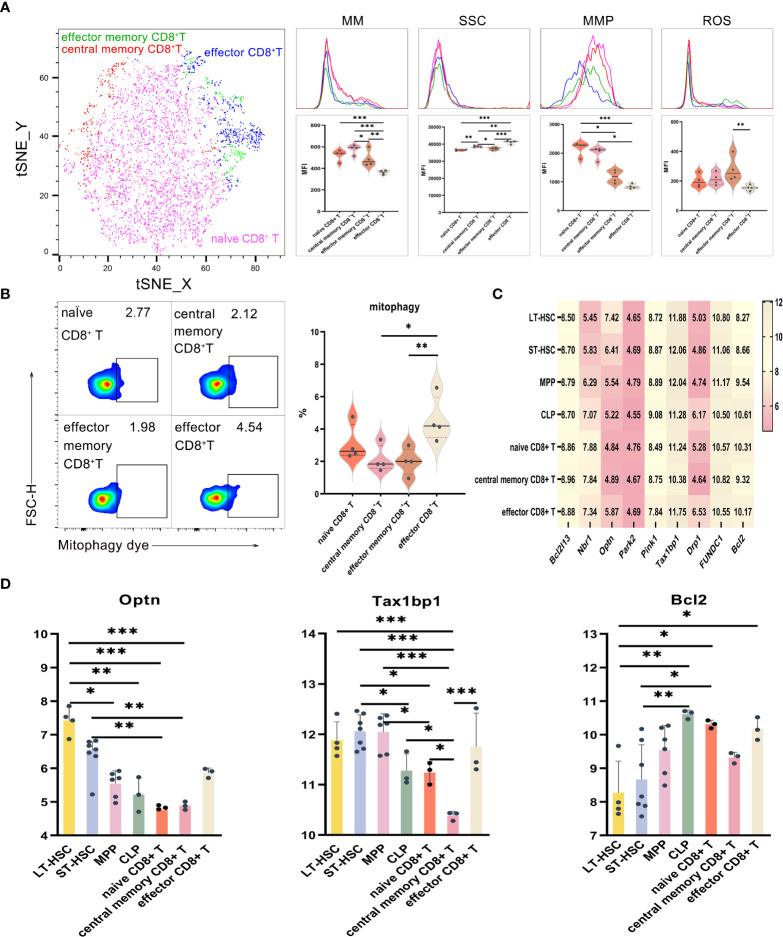
**(A)** t-SNE, MM, SSC, MMP and ROS in effector CD8^+^ T, CD8^+^ Tem and CD8^+^ Tcm **(B)** Mitophagy in effector CD8^+^ T, CD8^+^ Tem and CD8^+^ Tcm **(C)** Heat map for the expression levels of autophagy-related genes in CD8^+^ T cells at different differentiation and activation stages **(D)** Statistics for the expression level of autophagy-related genes. Normally-distributed data are presented as the mean ± SD, while non-normally-distributed ones are presented as the median with the interquartile range. One-way ANOVA or the Kruskal–Wallis test was used for comparisons and determining the *P* values. *P<0.05, **P<0.01, ***P<0.001.

UMAP, t-SNE and FlowSOM analyses showed that CD8^+^ T lymphocytes could be divided into subsets having different phenotypes. As described earlier, the levels of mitophagy, ROS, MMP and MM were analyzed to determine the mitochondrial functions of different CD8^+^ T cell subsets, with results for effector CD8^+^ T, CD8^+^ Tem and CD8^+^ Tcm shown in **Figures 5A, B**. When comparing activated subsets of CD8^+^ T cells, the MM levels were statistically different, with CD8^+^ Tcm having the highest levels, followed by naive CD8^+^ T, CD8^+^ Tem and effector CD8^+^ T eventually having the lowest ones. Hence, it was clear that relatively primitive subpopulations had higher MM in comparison with relatively differentiated ones. Regarding MMP levels, those of naive CD8^+^ T and CD8^+^ Tcm were significantly higher compared with CD8^+^ Tem, while the latter had significantly higher ROS levels compared with effector CD8^+^ T (**Figure 5A**). Finally, effector CD8^+^ T had a significantly higher mitophagy level in comparison with both CD8^+^ Tcm and CD8^+^ Tem, thereby showing that subsets of activated T cells displayed higher mitophagy (**Figure 5B**). Among the subsets of CD8^+^ T, the cells’ order in increasing levels of SSC were naive CD8^+^ T, CD8^+^ Tem, CD8^+^ Tcm and effector CD8^+^ T cells (*P* < 0.01), hence indicating that active cells were of greater complexity and had a higher number of organelles.

Statistical analyses of the expression of autophagy-related genes for CD8^+^ T cell at different differentiation and activation stages indicated that *Optn* was significantly more expressed in LT-HSC and ST-HSC compared with naive CD8^+^ T and CD8^+^ Tcm (*P* < 0.05), with CD8^+^ Tcm having a significantly lower level of *Tax1bp1* expression in comparison with LT-HSC, ST-HSC, MPP, CLP, naive CD8^+^ T and effector CD8^+^ T (*P* < 0.05). These results show that CD8^+^ Tcm inherited the least characteristics of HSC in terms of their participation in the Pink1/Park2 pathway, especially when compared with CLP and effector CD8^+^ T cells. For the BNIP3/NIX pathway, *Bcl2* was significantly more expressed in naive CD8^+^ T and effector CD8^+^ T cells compared with LT-HSC cells (*P* < 0.01), thus indicating that, in a similar way to CLP, the former was more involved in the BNIP3/NIX pathway (**Figures 5C, D**).

## Discussion

This exploratory work was focused on comparing the role of mitochondrial metabolism in mouse lymphocytic cells at different stages of their differentiation and activation. The heterogeneity of the mitochondrial autophagy pathway as inherited by lymphocyte subsets from hematopoietic stem cells was then discussed. The results first showed that of all lymphocytes, B cells had a relatively high mitochondrial metabolic activity and among their subsets, MM and MMP levels were relatively high in pro-B cells but mitochondrial metabolism level was relatively low for mature B cells. This could be due to the short survival cycle of mature B cells and the impairment of mitochondrial functions which lead to mitochondrial depolarization and a reduction in membrane potential, with the ultimate result being a change in cell membrane permeability and apoptosis (**Figure 3A**). Among the subsets of CD4^+^ T cells, a relatively high level of mitochondrial metabolism was noted for naive CD4^+^ T cells. In addition, CD8^+^ Tcm had relatively high levels of MM and MMP but relatively low levels of mitophagy, while effector T cells displayed opposite characteristics. These cells also show heterogeneity in the selection of FUNDC1, BNIP3/NIX and Pink1/Park2 pathways for mitochondrial autophagy. In this context, B cells showed greater similarity to LT-HSC and ST-HSC in terms of their participation in the Pink1/Park2 pathway as well as for inheriting most of the autophagy pathway characteristics of HSC. On the other hand, among the subsets of lymphocytes, pro-B, nT_reg_ and CD8^+^ Tcm inherited the least characteristics of HSC, especially in terms of their participation in the Pink1/Park2 pathway.

Mitochondria are dynamic organelles which can change their shapes and positions within cells, through coordinated cycles of division and fusion, to regulate their own functions and cell metabolism. Mitochondria can also coordinate immunity by regulating the metabolism and physiological status of different types of immune cells. For instance, they can regulate the development, activation, proliferation, differentiation and death of the cells. In addition, mitochondria have cell specificity, and since their protein expression can be different in different cells, the metabolic functions of mitochondria in different types of cells can also vary. Mitochondria with complete structures and normal functions are essential organelles to maintain the stability of hematopoietic cells. In lymphocytes, B cells have the highest levels of MM and ROS, and these not only reflect the high level of mitochondrial metabolism of B cells but also lead to an easier accumulation of mitochondria in B cells. When mitochondria are damaged, excessive pressure signals are generated to induce cell damage which eventually leads to programmed cell death. Given that changes in mitochondrial functions due to oxidative phosphorylation injury, abnormal energy metabolism, inhibition of apoptosis, autophagy disorder, promotion of immune escape and changes in signal pathways, can influence the occurrence of diseases, mitochondrial autophagy actually represents a cell defense mechanism which selectively removes aging and damaged mitochondria to promote mitochondrial renewal and maintain mitochondrial functions. Therefore, for B cells, autophagy is an effective mechanism to protect cells. Weak mitophagy and high intracellular ROS levels are not conducive to the maintenance of cellular functions and are prone to induce apoptosis.

In lymphocytes, the levels of MM, MMP and ROS of CD4^+^ T cells were significantly higher than those of CD8^+^ T cells (*P*<0.05), reflecting the strong mitochondrial functions of CD4^+^ T cells. This phenomenon may be related to a change in the mitochondrial content of T cells during lineage development. During the process of differentiation, T cells turn into a metabolically active state characterized by “increased nutrient intake, increased glutamine decomposition and increased glycolysis metabolism” to meet the demand for active proliferation. T cells also increase the mitochondrial pool, with the mitochondrial content increasing accordingly. In the study of immune cells, it was found that ROS played a very important role in the process of T cell differentiation. The high concentration of ROS in the environment was conducive to the production of IL-2 and IL-4, promoting Th2 differentiation and prolonging Th2-mediated immune response. The corresponding low concentration of ROS promoted the differentiation of Th1 and Th17. In addition, some studies also showed that in CD8^+^ T cells, low MPP showed enhanced persistence *in vivo* compared with high MMP. By activating CD8^+^ T cells, the proliferation resulting from low membrane potential is less than that induced by CD8^+^ T with high membrane potential. CD8^+^ T cells can also enhance autoimmunity and greater anti-tumor effects. The increase of MMP is helpful to promote the secretion and function of effector cytokines in various lymphocyte lineages. For example, in CD8^+^ T cells, high MPP displayed increased oxidative stress, DNA damage response, cell cycle arrest and T cell failure, which altogether may lead to T cell apoptosis. On the other hand, low MMP is more effective in combating oxidative stress, protecting against DNA damage and resisting apoptosis. Therefore, the differences between CD4^+^ T cells and CD8^+^ T cells at MM, MMP and ROS levels may indicate the heterogeneity of lineage development (16–20).

The immune system is very important for resisting infections and tumors. It consists of an adaptive and innate system, with the former involving T and B lymphocytes. This study found that the ROS levels of CD4^+^ T and CD8^+^ T cells were significantly lower compared NK cells (**Figure 3A**), and this could be due to the fact that cells of the adaptive immunity do not rely mainly on ROS to perform effector functions. At the same time, low levels of physiologically-related ROS are already produced during the process of T cell development and activation (21). Additionally, a significantly higher level of mitochondrial metabolism was observed for naive CD4^+^ T cells in comparison with nT_reg_ and memory CD4^+^ T cells (**Figure 4A**). These differences could be attributed to the low nutrient absorption rate of naive T cells in a resting state as their survival depends on the amount of ATP generated by oxidative phosphorylation, driven by the oxidation of glucose, glutamine and fatty acids (22). However, among the subset of CD8^+^ T cells, effector CD8^+^ T cells had the lowest level of mitochondrial metabolism (**Figure 5A**). This could have been because T cell activation results in an increased uptake of glucose in effector T cells in order to carry out glycolysis for the rapid production of ATP and for maintaining cellular functions through higher mitophagy levels (19, 23, 24). Effector T cells depend on glycolysis due to its link to mTOR activation. Indeed, mTOR activation assist in development and differentiation of effector T cell subsets (25–27). T_reg_ cells can have regulatory functions through its inhibitory effects on other immune cells and as such, they can control both adaptive and innate immune responses. However, instead of glycolysis as it is the case for effector T cells, oxidative phosphorylation as well as fatty acid oxidation are key for the differentiation of T_reg_ cells and for maintaining their immunomodulatory functions (28, 29).

The adaptive immunity is characterized by its ability to produce long-lived memory cells. After an adaptive immune response, especially in the later stages of immune effect, most effector T and effector B cells undergo apoptosis. However, some differentiate into antigen-specific memory cells which are preserved so that they can rapidly proliferate under restimulation by an antigen, thus providing immune memory protection for the body (30, 31). The role and high heterogeneity of T cells in humoral and cellular immune responses are much greater than for B cells. For instance, the ROS levels of CD8^+^ Tcm were lower compared with CD8^+^ Tem. This could be due to decreased glycolysis during the development of memory T cells as well as the fatty acid oxidation in mitochondria to promote metabolism (**Figure 5A**). This enhanced mitochondrial metabolism could be necessary to accelerate memory cells proliferation in response to restimulation by homologous antigens.

The two subpopulations of memory T cells namely effector memory T cells (Tem) and central memory T cells (Tcm) not only participate in the maintenance of an immune cell bank, but also play an important role in immune response (32). Among these, TEM mediates protective memory by migrating to peripheral inflammatory sites where they play effector functions. TCM, on the other hand, is involved in reactive memory. It is present in the T cell area of secondary lymphoid organs, and even though it has practically no effector functions, stimulation by an antigen can induce its proliferation and subsequent differentiation into effector cells (33). Further comparisons show that, unlike TCM, TEM can rapidly perform effector functions to produce interferons under the stimulation of antigen- γ, interleukin-4 and interleukin-5, amongst others. Furthermore, while TCM is highly sensitive to antigen stimulation and can, to a certain extent, differentiate into TEM and short-term effector T cells, TEM can only differentiate into short-term effector T cells. Finally, compared with the latter, memory T cells have a greater mitochondrial network with an increased number of mitochondria that can display standby respiratory capacity (**Figure 5A**). As far as NK cells are concerned, being an important component of the innate immune system, they can upregulate glycolysis levels during activation and proliferation in response to increased cellular demands for nucleotides, lipids and amino acids.

Studies have shown that there is a dynamic balance in the MM of cells, with mitochondrial distribution, arrangement, numbers, size and morphology being different in cells which are at different stages of differentiation and activation (34–36). MMP is the mitochondrial transmembrane potential difference formed when energy generated by the tricarboxylic acid cycle is transferred to electrons. This potential difference pumps protons from the matrix side of the mitochondrial inner membrane to the outside of the inner membrane while the electrons are transmitted through the respiratory chain (37, 38). Another feature of mitochondrial function which was considered in this study is ROS which represents an inevitable product of cell metabolism. High levels of ROS in cells can directly or indirectly participate in cell signal transduction and induce apoptosis, with this process being common to tumors and many other diseases (39, 40). However, it should be noted that while ROS are mainly produced in mitochondria, the latter are also highly sensitive to ROS which tend to induce oxidative damage through the oxidation of mitochondrial cardiolipin, DNA as well as other important proteins, before ultimately leading to apoptosis (41–43). Under these conditions, mitochondria can undergo autophagy whereby the cells selectively carry out the isolation and degradation of incomplete or damaged mitochondria in order to maintain cellular homeostasis as well as the integrity of the mitochondrial network (44, 45). Therefore, levels of mitochondrial autophagy, ROS, MMP and MM were selected as the main parameters for evaluating the mitochondrial functions of lymphocytes.

Previous studies have shown that in comparison with other types of hematopoietic cells, HSCs have relatively low levels of mitochondrial ROS, MMP and MM but relatively higher ones for mitophagy. These differences could help to maintain the self-renewal ability of HSC. Indeed, mitophagy regulates the levels of MM and ROS in cells, and as such, it could be a means of regulating the early directional differentiation of HSC. Furthermore, compared with the other lymphocytes, B cells, especially pre-B and immature B cells, inherited more metabolic characteristics from HSC in terms of mitophagy quantity and pathway selection. Compared with hematopoietic stem progenitor cells, there was also greater heterogeneity in the mitochondrial metabolism and other functions of lymphocytes. This heterogeneity in lymphocyte functions is not only reflected at different differentiation and activation stages, but also within the same lymphocyte population. The results showed that the morphological and functional heterogeneity of innate immune cells (NK) was stronger than for adaptive immune cells (B, CD8^+^ T and CD4^+^ T). In B, CD8^+^ T and CD4^+^ T subpopulations, pro-B, nT_reg_ and effector CD8^+^ T cell populations displayed greater heterogeneity than other cells within the same subpopulation (**Figure S4**). The presence of this heterogeneity suggests potential functional differences, including in mitochondrial function, between single cells of the same immunophenotype. However, such differences are likely to be missed in single-cell research. Therefore, compared with the studies involving single cells, studying cellular functions based on flow cytometry may better reflect the functional characteristics of a lymphocyte population.

Lymphoid hematopoietic cells are considered to be a heterogeneous cell population and they are largely involved in immune functions. Mitochondrial autophagy is an important mechanism that allows cells to regulate and maintain mitochondrial homeostasis for ensuring their quality and functions and this process is precisely regulated by over 30 autophagy-related genes. The heterogeneous nature of mitochondrial autophagy is important for the differentiation and activation of lymphoid hematopoietic cells. In fact, the differential expression of lineage-related genes in lymphoid hematopoietic cells leads to significant differences in the transcription levels of these cells. Similarly, as far as autophagy-related genes are concerned, additional analysis of single-cell RNA-Seq data also indicated differences in their transcription levels between different populations of lymphoid hematopoietic cells. These differences can provide a better understanding of the heterogeneity of mitochondrial autophagy in lymphocytes at different differentiation and activation stages. Furthermore, based on single-cell RNA-Seq technology, the typical characteristics of different types of lymphoid hematopoietic cells can be determined, especially in terms of inherited mitochondrial autophagy functions.

In the bone marrow, lymphocytes first produce juvenile lymphoid progenitor cells which then migrate to peripheral lymphoid organs such as lymph nodes and spleen where they mature. So, there are more primitive lymphocytes (pro B cells, T lymphoid progenitor cells, etc.) in the bone marrow, while lymph nodes and spleen, which are sites of immune response, have a higher proportion of mature lymphocytes (mature B cells, CD4^+^ T cells, CD8^+^ T cells, etc.). In the process of immune response, the energy supply pathway of lymphocytes changes, and the morphology as well as dynamics of mitochondria also change (11, 46). Compared with the spleen and lymph nodes, the lymphocytes in the bone marrow are mainly lymphoid progenitor cells such as pro B cells, naive CD4^+^ T cells and naive CD8^+^ T cells. The mitochondria of these juvenile lymphocytes are mostly small and round, with the mitochondrial membrane potential being low. In addition, the synthesis of complex I and III, which are the production sites of reactive oxygen species in the electron transfer chain, is reduced. Therefore, the ROS produced by the mitochondria of lymphocytes in the bone marrow is less than that in the spleen and lymph nodes, with the energy metabolism being mainly oxidative phosphorylation. In contrast, there is a higher proportion of mature B cells, effector CD8^+^ T cells, memory T cells and CD4^+^ T_reg_ cells in the spleen and lymph nodes. The mitochondria of these cells have a higher total mass and an elongated oval shape. Moreover, their energy metabolism is dominated by the aerobic glycolysis of lactic acid to supply the energy needed for the cellular immune response process. There is also higher production of ROS as well as more frequent mitochondrial autophagy to ensure sufficient substrate and energy for the repair of mitochondrial damage (1, 19, 46, 47).

Antigen presentation refers to the process during which an antigen is ingested and processed by antigen presenting cells (macrophages, dendritic cells, etc.), before being presented on the cell surface in the form of MHC complex and recognized by immunocompetent lymphocytes. After recognition and binding of antigen peptides with BCR, TCR and other lymphocyte surface receptors, a series of signal transduction cascades occur in the lymphocytes, and these ultimately enable lymphocytes to undergo appropriate biological changes for immune response. During the immune response, different lymphocytes play different biological functions, and this requires specific changes in the mitochondrial functions of different types of lymphocytes. T cells differentiate into CD4^+^ T cells and CD8^+^ T cells through single positive selection, with CD8^+^ T cells being mainly involved in the killing of target cells. During the process of transforming into effector T cells, the metabolic mode of cells changes significantly. Mitochondria, as the center of anabolism, also change significantly with the change in cell state. For instance, in terms of shape, they extend from round to oval, with more internal folds, more DNA synthesis and higher membrane potential. Furthermore, their metabolism change to the aerobic glycolysis of synthetic lactic acid, with more ROS production and more mitochondrial autophagy so that cells can rapidly proliferate and kill target cells (19).

Mitochondrial metabolic functions determine the lineage differentiation of CD4^+^ T helper cells which is mainly regulated by the generation of ROS necessary for NFAT, NF-kB and proximal TCR signal transduction. For example, compared with Th17 cells, the oxidative phosphorylation level of T_reg_ increases and the glycolytic flux decreases. Similar to T cells, B cells also experience a major transformation in their metabolic mode as they switch from their initial resting state to proliferating cells. After binding with antigens, B cells greatly enhance glucose and glutamine metabolism during cloning and amplification. BCR stimulates the release of calcium ions into the cytoplasm, promotes the production of ROS in mitochondria and allows the activation of downstream signal pathways. In this case, the level of ROS production affects the activation of downstream signaling pathways. During antigen presentation, NK cells promote various biological processes at the molecular level by upregulating glycolysis and oxidative phosphorylation. Generally speaking, resting mature NK cells maintain their cell homeostasis mainly through oxidative phosphorylation. When they are affected by proinflammatory cytokines such as IL2 and IL12, they display a strong upregulation of glycolysis and oxidative phosphorylation pathways (47–49).

BNIP/NIX is a protein containing only the BH3 domain family which regulates mitochondrial autophagy and controls the change of total mitochondrial mass during the immune response of lymphocytes (50). FUNDC1 protein can interact with DNM1L/DRP1 and OPA1 proteins to regulate mitochondrial fusion and division, while also regulating mitochondrial autophagy. Therefore, it can couple changes in mitochondrial dynamics with mitochondrial quality control (51). Pink/Part2 is also related to the morphological and dynamic changes of mitochondria (52). When lymphocytes are in a resting state and change into a proliferating state, their mitochondria play a synergistic role through the above three ways to promote the division of the lymphocytes’ mitochondria. The total mass of the mitochondria then increases, their membrane potential increases and their inner folds also increase so as to carry out more biochemical reactions that provide the energy required by the lymphocytes for the immune response. At the same time, they also generate more ROS or act as a second messenger, with increased mitochondrial autophagy promoting the repair of mitochondrial damage.

One limitation of this study was that instrument limitations as well as the conditions for matching antibodies made it difficult to carry out an in-depth analysis of the mitochondrial metabolism of all types of lymphoid and myeloid hematopoietic cell subsets. Future works are expected to include subsets of myeloid hematopoietic cell to explore the mitochondrial functions in monocyte/macrophage subsets (classical and non-classical monocytes/macrophage), granulocyte subsets (myeloblast, promyelocyte, myelocyte and metayelocyte) and erythroid cell subsets (BFU-E, CFU-E, proerythroblasts and polychromatic erythrocytes). This will add to the current understanding of the mitochondrial functions of hematopoietic cells.

The heterogeneity of mitochondrial functions are important for lymphoid hematopoietic differentiation. In this work, to ensure uniformity, each mitochondrial feature was detected by using the same flow cytometer at the same time. The voltage of the photomultiplier tube of the target fluorescence channel was also consistent in each flow template, with potential interference from other fluoresceins on the target fluorescence channel eliminated by regulating fluorescence compensation regulation. In this way, the mitochondrial functions of 14 main types of lymphocytes were determined at different differentiation and activation stages simultaneously. This study compared the mitochondrial metabolism of different lymphocytes and their subsets to provide a complete set of methods and standards for analyzing the mitochondrial mitophagy, ROS, MMP and MM levels of hematopoietic cells, Furthermore, mitochondrial metabolic maps of lymphocytes were drawn at different stages of their differentiation and activation while single cell sequencing technology allowed the heterogeneity of the mitochondrial autophagy pathway, as inherited from lymphoid hematopoietic cells, to be analyzed. The current results provide a standard and reference for future studies involving mitochondrial metabolism in subsets of myeloid cells, especially since these functions are also closely related to the maintenance of hematopoietic homeostasis. Finally, this study may serve as a reference in the study of tumor and aging-related biomarkers based on mitochondrial metabolism.

## Conclusions

In this work, the mitochondrial functions of lymphocytes were compared at different differentiation and activation stages and included CD8^+^ T lymphocytes, CD4^+^ T lymphocytes, B lymphocytes, NK cells as well as their subsets. A complete set of methods was used to comprehensively analyze mitophagy levels, ROS, MMP and MM of subsets of lymphocytes. Of all lymphocytes, B cells had a relatively high mitochondrial metabolic activity which was evident from the higher levels of mitophagy, ROS, MMP and MM, and this reflected the highly heterogeneous nature of the mitochondrial metabolism in lymphocytes.

Meanwhile, the autophagy-related genes of lymphoid hematopoietic cells including hematopoietic stem cells, hematopoietic progenitor cells and lymphocyte subsets were analyzed, which preliminarily showed that these cells were heterogeneous in the selection of mitophagy related Pink1/Park2, BNIP3/NIX and FUNDC1 pathways. The results showed that compared with CD4^+^ T, CD8^+^ T and NK cells, B cells were more similar to LT-HSC and ST-HSC in terms of their participation in the Pink1/Park2 pathway, as well as the degree to which the characteristics of autophagy pathway were inherited from HSC.

## Data availability statement

The original contributions presented in the study are included in the article/**Supplementary Material**. Further inquiries can be directed to the corresponding authors.

## Ethics statement

The animal study was reviewed and approved by the Institutional Animal Care and Use Committees of the State Key Laboratory of Experimental Hematology.

## Author contributions

HL, WF, and WY contributed equally to this work. YZ, YG, and XK conceived and directed the project; HL and WF performed the experiments; HL, WF, WY, ZC, XK, and EL analyzed the data; HL wrote the manuscript. WF, WY, ZC, XK, EL, FS, YG, and YZ contributed to the discussions and comments on the paper. All authors contributed to the article and approved the submitted version.

## Funding

This work was supported by grants from National Natural Science Foundation of China (NSFC 81970120, 81870083 and 81970105), Tianjin Science and Technology Planning Project (No. 18ZXXYSY00010) and CAMS Innovation Fund for Medical Sciences (2022-I2M-2-003). This work was supported funded by Tianjin Key Medical Discipline (Specialty) Construction Project (No. TJYXZDXK-006A).

## Acknowledgments

All authors kindly thank the core facilities of State Key Laboratory of Experimental Hematology, Institute of Hematology & Blood Diseases Hospital, Chinese Academy of Medical Sciences & Peking Union Medical College for the technical assistance.

## Conflict of interest

The authors declare that the research was conducted in the absence of any commercial or financial relationships that could be construed as a potential conflict of interest.

## Publisher’s note

All claims expressed in this article are solely those of the authors and do not necessarily represent those of their affiliated organizations, or those of the publisher, the editors and the reviewers. Any product that may be evaluated in this article, or claim that may be made by its manufacturer, is not guaranteed or endorsed by the publisher.

## Abbreviations

LT-HSC, long-term hematopoietic stem cell; ST-HSC, short-term hematopoietic stem cell; ROS, reactive oxygen species; MMP, mitochondrial membrane potential; MM, mitochondrial mass; NK, Natural Killer; HSC, hematopoietic stem cell; MPP, multipotent progenitor cell; CLP, common lymphoid progenitor cell; PBS, phosphate buffer saline; SSC, side scatter characteristics; SD, standard deviation; t-SNE, t-distributed stochastic neighbor embedding; UMAP, uniform manifold approximation and projection; Tem, effector memory T cell; Tcm, central memory T cell.
