# Supplementary material for: Elucidating the mitochondrial function of murine lymphocyte subsets and the heterogeneity of the mitophagy pathway inherited from hematopoietic stem cells
Source: Front Immunol. 2022 Nov 7;13:1061448. doi: 10.3389/fimmu.2022.1061448 (PMC9676649; doi:10.3389/fimmu.2022.1061448)
Supplement: Supplementary file 1 [file DataSheet_1.docx]

Supplementary Material

**Supplementary Figure Captions**

**Figure S1.** Differentiation and activation of hematopoietic cells

**Figure S2.** Flow cytometry plot of 15 populations of lymphocytes isolated from the bone marrow of mice

**Figure S3.** Clustering results of UMAP and FlowSOM for populations of mouse lymphocytes

**Figure S4.** Heterogeneity of lymphocyte populations at the same differentiation stage

**Supplementary table captions**

**Table S1.** Immunophenotypes of lymphocytes

**Table S2.** Antibody combinations for mitochondrial mass detection (MitoTracker Green)

**Table S3.** Antibody combinations for mitochondrial membrane potential detection (MitoTracker Red)

**Table S4.** Antibody combinations for mitochondrial reactive oxygen species detection (MitoSOX Red)

**Table S5.** Antibody combinations for mitophagy detection (Mitophagy Dye)

**Table S6.** Information on the antibodies


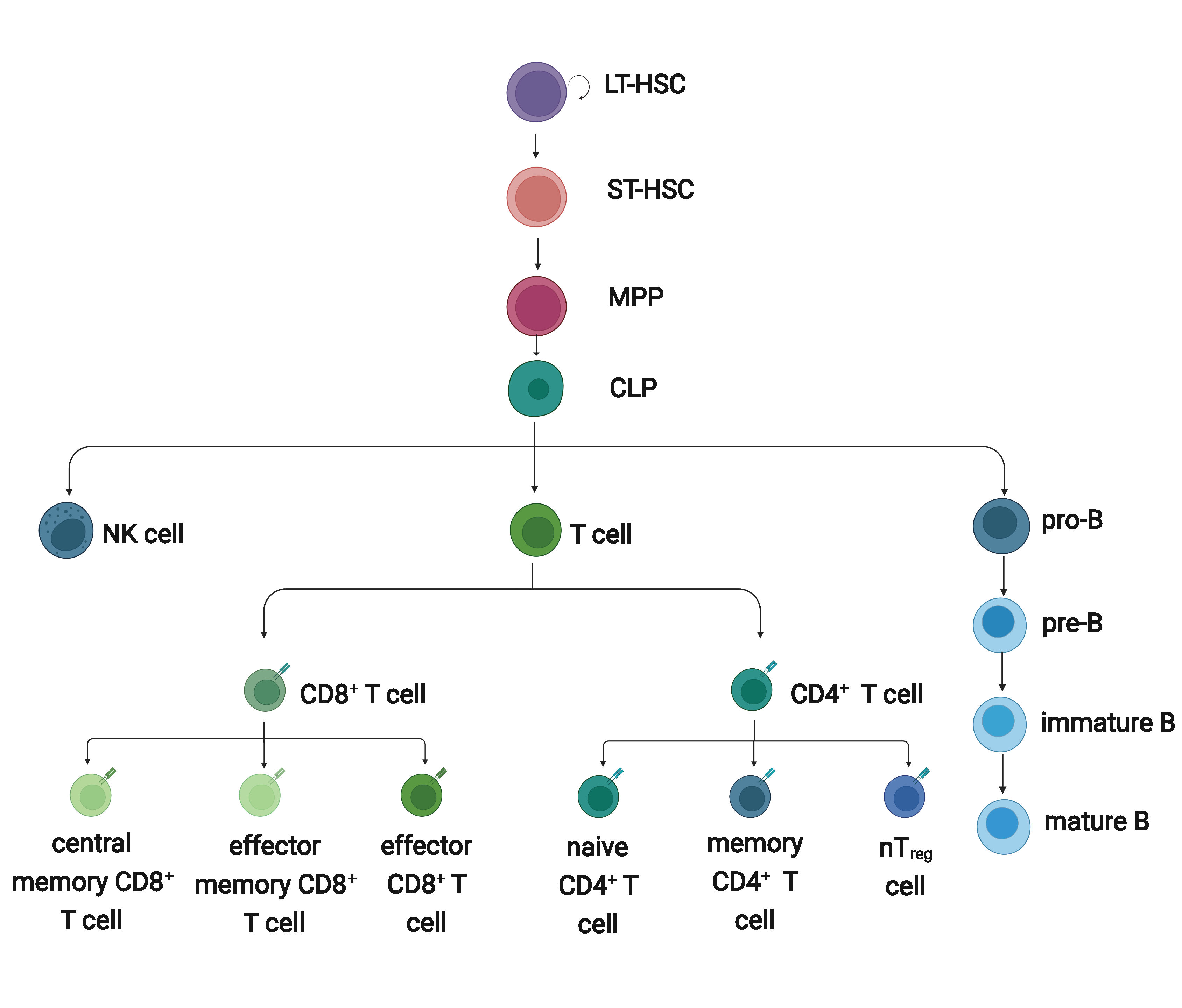


**Figure S1.** Differentiation and activation of hematopoietic cells


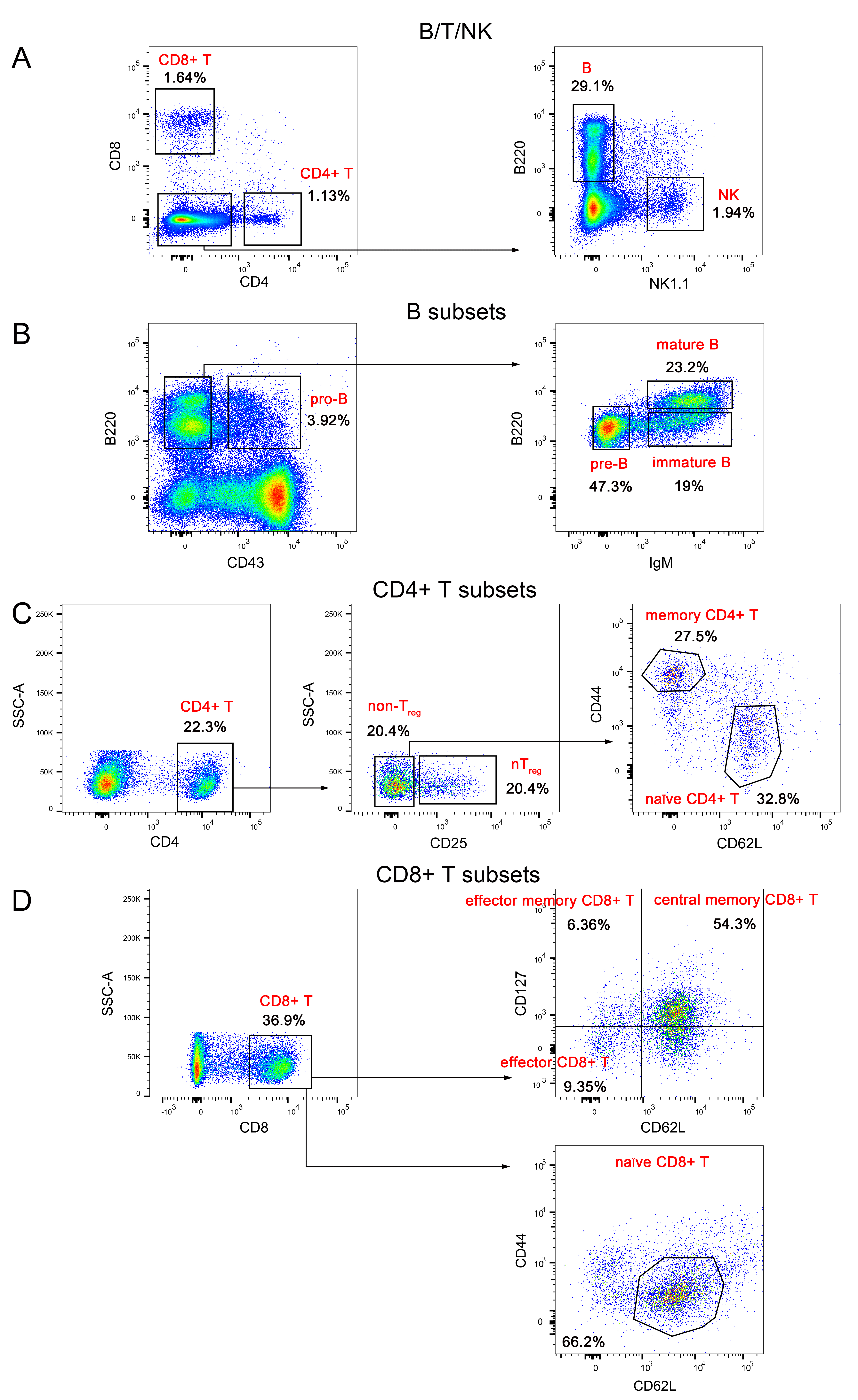


**Figure S2.** Flow cytometry plot of 15 populations of lymphocytes isolated from the bone marrow of mice. A.B, CD4^+^ T, CD8^+^ T and Natural Killer cell (NK) populations; B. pro-B, pre-B, immature B and mature B populations; C. naive CD4^+^ T, memory CD4^+^ T and nT_reg_ populations; D. naive CD8^+^ T, CD8^+^ Tcm, CD8^+^ Tem and effector CD8^+^ T populations.


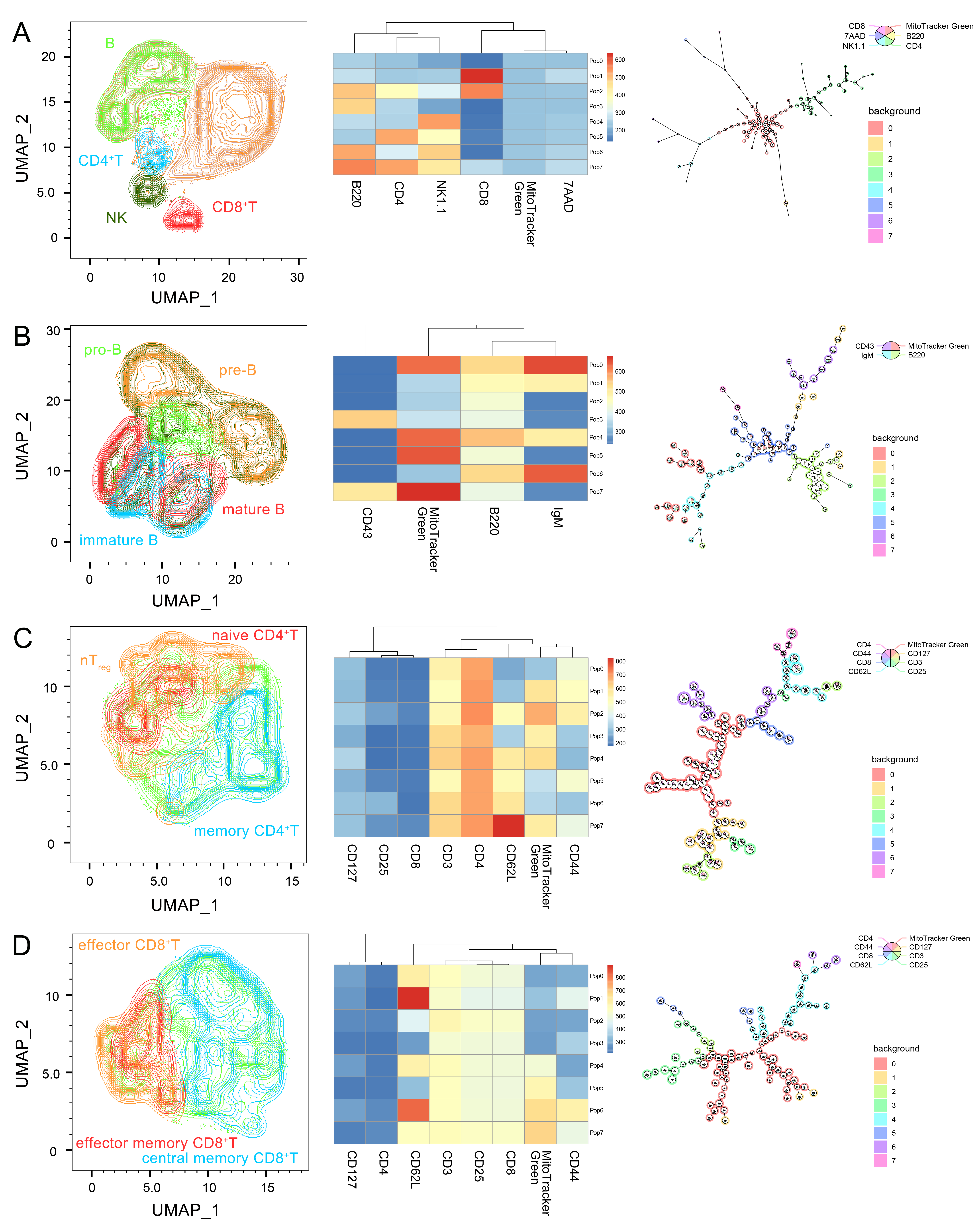


**Figure S3.** UMAP, FlowSOM heatmap and FlowSOM cluster analysis of 14 populations of lymphocytes. A. B, CD4^+^ T, CD8^+^ T and NK populations B. subsets of B cells C. subsets of CD4^+^ T D. subsets of CD8^+^ T.

In order to study the relationship between the characteristics of differentiated lymphocyte populations and mitochondrial functions, a unified manifold approximation and projection (UMAP) as well as a flow self-organizing feature map (FlowSOM) were used to characterize the populations at different differentiation and activation stages. UMAP analysis can identify the high-dimensional protein marker correlation, visualize the high-dimensional similarity of cells, and provide a new method for the analysis of flow cytometry data. Results of the UMAP analysis reflect the antigen expression and distribution of the lymphocytes B, CD4^+^ T, CD8^+^ T and NK and their subsets pro-B, pre-B, immaturity B, mature B (B subsets), naive CD4^+^ T, memory CD4^+^ T, nT_reg_ (CD4^+^ T subsets), CD8^+^ Tcm, CD8^+^ Tem and effector CD8^+^ T (CD8^+^ T subsets) (Figures S3A-S3D). The results were consistent with the proportion of cell population obtained by classical two-dimensional flow, step-by-step circle gate.

As shown in Figure S3A, the BTNK cell population was divided into eight groups by FlowSOM. The color density of the heat map represents the average expression of a given antigen, with the heat map generated after normalization. The heatmap also shows the median expression intensity of each protein marker of each detected cell population, and clusters are grouped based on differences in the intensity of protein expression. Before presenting the data, the sample was set to FlowSOM and the live cell flow gate was manually circled on the FSC-SSC scatter diagram. In this study, only the cells in the living cell gate were extracted and FlowSOM was used for further analysis. Six surface markers were used in the analysis, and the color of the grid represents the intensity of fluorescent markers. The clusters Pop3, Pop5, Pop1 and Pop4, assigned according to protein expression in the grid, correspond to B, CD4^+^ T, CD8^+^ T and NK cell populations in the classical flow cytometry, respectively. FlowSOM provides a means of visually representing cell population cluster analysis by characterizing antigen expression as well as the distribution of cells (represented as nodes) based on node color and the relative position between the nodes. The larger the fan-shaped area of the node, the stronger the expression of the antigen. Subsets of B cells were divided into eight groups by FlowSOM. The clusters Pop7, Pop2, Pop0+Pop1+Pop6 and Pop4, assigned according to protein expression, correspond to pro-B, pre-B, immature B and mature B cell populations in the classical flow cytometry, respectively (Figure S3B). Similarly, CD4^+^ T subsets were divided into eight groups by FlowSOM. The clusters Pop7, Pop1 and Pop2+Pop6, assigned according to protein expression, correspond to naive CD4^+^ T, memory CD4^+^ T and nT_reg_ cell populations in classical flow cytometry, respectively (Figure S3C). Finally, CD8^+^ T subsets were divided into eight groups by FlowSOM, with the clusters Pop4+Pop6, Pop2 and Pop3+Pop5, assigned according to protein expression, corresponding to CD8^+^ Tcm, CD8^+^ Tem and effector CD8^+^ T cell populations in the classical flow loop gate method, respectively (Figure S3D). UMAP and FlowSOM analyses showed that lymphocytes, at different differentiation and activation stages, could be subdivided based on different phenotypes. These results provide a foundation for studying the mitochondrial functions of lymphocytes at different differentiation and activation stages.


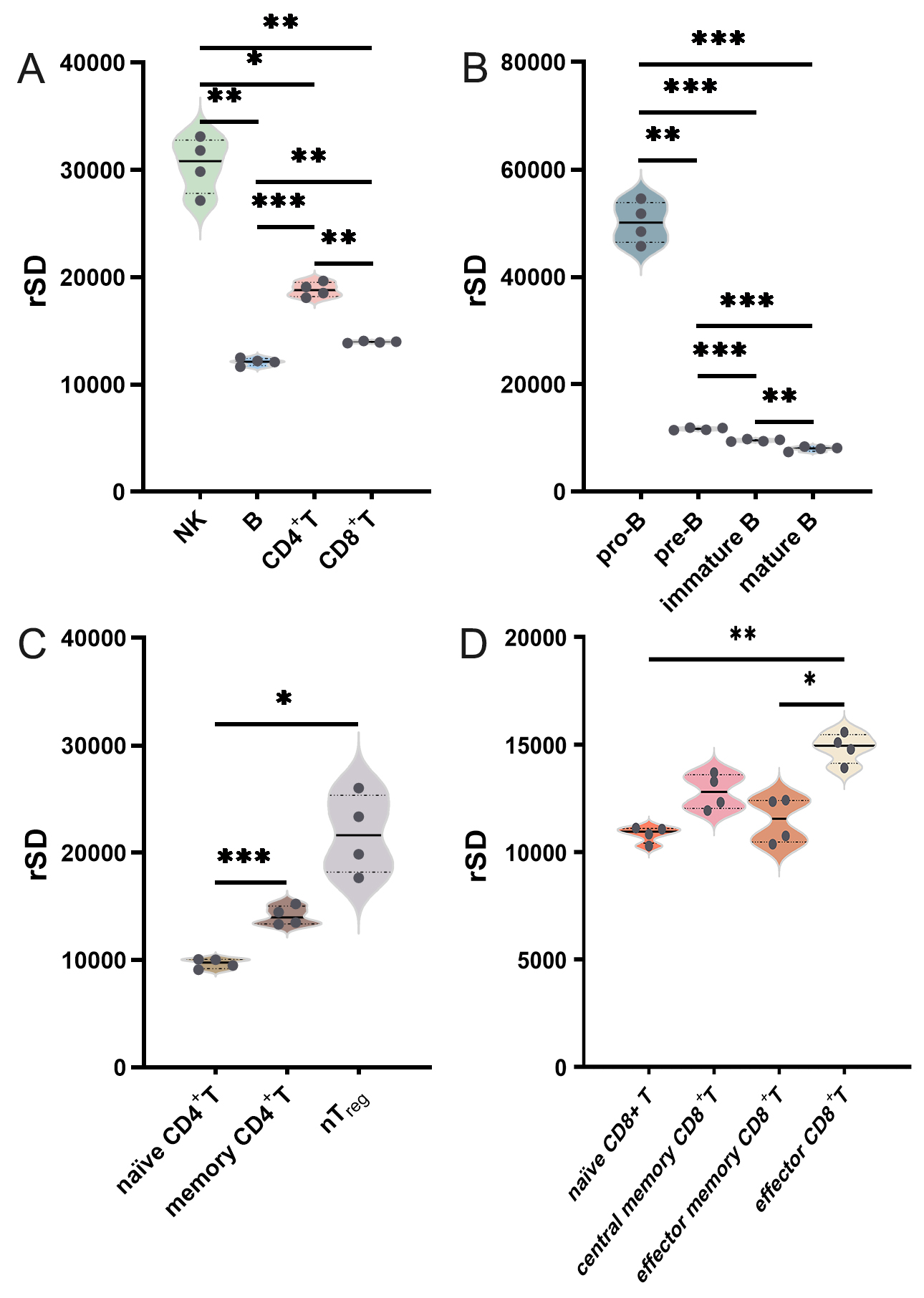


**Figure S4.** Relative standard deviation (rSD) A. B, CD4^+^ T, CD8^+^ T and NK populations B. subsets of B cells C. subsets of CD4^+^ T D. subsets of CD8^+^ T. Normally-distributed data are expressed as the mean ± SD. *P*-values were determined by one-way ANOVA. **P*<0.05, ***P*<0.01.

After the study of the mitochondrial functions of lymphocytes at different differentiation and activation stages, in order to explore the functional heterogeneity of lymphocytes at similar stages based on the classification of cell surface markers, flow cytometry was used for further analysis, while SSC-based rSD was used to evaluate the degree of difference between the components within a cell population. Figure S4 shows the rSD (SSC) results of lymphocytes at different differentiation and activation stages, the rSD (SSC) values of lymphocytes as well as the results for B subsets, CD4^+^ T subsets and CD8^+^ T subsets. Overall, the rSD (SSC) levels for the four types of cells in decreasing order were NK cells, CD4^+^ T cells, CD8^+^ T cells and B cells and these values were consistent with the SSC results of the four types of cells. Specifically, in lymphocytes, the rSD (SSC) level of NK cells was significantly higher than that of the other three groups of cells (P < 0.001), and the rSD (SSC) level of B cells was significantly lower than that of the other three groups of cells (P < 0.001) (Figure S4A). These results show that compared with other lymphocytes, the distribution of SSC in B cell populations deviates from the average value and has low heterogeneity, while NK cells have the opposite result. In subsets of B cells, the rSD level of poorly differentiated pro-B cells was significantly higher than that of pre-B, immature B and immature B cells (P < 0.001), which was consistent with the results of SSC (Figure S4B). In subsets of CD4^+^ T, the rSD level of naive CD4^+^ T was significantly lower than that of memory CD4^+^ T and nT_reg_ cells (P < 0.001), which was consistent with the results of SSC (Figure S4C). In subsets of CD8^+^ T, the rSD level of effector CD8^+^ T was significantly higher than that of naive CD8^+^ T and CD8^+^ Tem (P < 0.05), which was consistent with the results of SSC (Figure S4D). The rSD changes of different types of lymphocytes are consistent with their SSC characteristics, indicating that the complexity of organelles was positively correlated with cell heterogeneity. The greater the number of organelles in cells, the more discrete the cell population is in the results of flow cytometry.

The heterogeneity of mitochondrial functions in lymphocytes at similar differentiation and activation stages indicates that there were limitations in characterizing the function of lymphocytes by cell phenotypic markers. Morphological and functional heterogeneity were also observed between cells within the lymphocyte population and with similar cell phenotypes. This result shows that the study of cell population may better reveal the functional characteristics of a class of lymphocytes than single-cell study. Therefore, rSD and other indicators provide ideas and methods for studying the function of organelles including mitochondria of lymphocytes at the same differentiation and activation stage.

**Supplementary Methods**

**MM detection**

The whole bone marrow cells of 2 C57 mice were taken, and the leukocytes were obtained with erythrocyte lysate. For cell counting, all cells were divided into 19 tubes which included 1 tube as the blank group, 1 tube in CD44 (BV605), 1 tube in CD62L (BV510), 1 tube in mitotracker green (MM), 4 tubes in B cell sample group (4 secondary holes), 4 tubes in T cell sample group (4 secondary holes), 4 tubes in NK cell sample group (4 secondary holes), 1 tube in T cell FMO group, 1 tube in B cell FMO group and 1 tube in NK cell FMO group. Cell concentrations of each tube were adjusted to 5x10^6^ cells/ml before adding antibody combinations for B, T and NK cells to their corresponding tubes (Table S2). Tubes were incubated at 4 °C for 30 min and after adding 3 ml of PBS, centrifugation was carried out at 1500 rpm for 5 min before discarding the supernatant. Then, 500 μl of Mitotracker Green working solution (100 nM) was added to the Mitotracker Green single positive tube and the full positive sample tubes. After incubating at 37 °C for 20 min and in the dark, 3 ml of PBS was added, the tubes were centrifuged at 1500 rpm for 5 min, and the supernatant was discarded. After repeating this washing step twice, the final pellet was resuspended in 500 μl of PBS, and the suspension was filtered through a 30–70μm nylon mesh. Finally, 3 μl of 7-AAD staining solution was added to each tube prior to analysis within 1 h.

**MMP detection**

The whole bone marrow cells of 2 C57 mice were taken, and the leukocytes were obtained with erythrocyte lysate. For cell counting, all cells were divided into 19 tubes and included 1 tube as the blank group, 1 tube in CD8 (BV650), 1 tube in CD25 (APC), 1 tube in mitotracker red (MMP), 4 tubes in B cell sample group (4 secondary holes), 4 tubes in T cell sample group (4 secondary holes), 4 tubes in NK cell sample group (4 secondary holes), 1 tube in T cell FMO group, 1 tube in B cell FMO group and 1 tube in NK cell FMO group. Cell concentrations of each tube were then adjusted to 5x10^6^ cells/ml before adding antibody combinations for B, T and NK cells to the corresponding cell tube respectively (Table S3). Tubes were incubated at 4 °C for 30 min and after adding 3 ml of PBS, centrifugation was carried out at 1500 rpm for 5 min before discarding the supernatant. Then, 500 μl of Mitotracker Red working solution (30 nM) was added to the Mitotracker Red single positive tube and full positive sample tubes. After incubation for 20 min, at 37 °C and in the dark, the subsequent steps were conducted as mentioned in section MM detection. The voltage of the side scatter light (SSC) in all flow cytometry templates was kept consistent, so that the relative standard deviation (rSD) of SSC signals could be calculated.

**Mitochondrial ROS detection**

The whole bone marrow cells of 2 C57 mice were taken, and the leukocytes were obtained with erythrocyte lysate. For cell counting, all cells were divided into 24 tubes and included 1 tube as the blank group, 1 tube in CD44 (BV605), 1 tube in CD62L (BV510), 1 tube in mitosox red (ROS), 4 tubes in B cell sample group (4 secondary holes), 4 tubes in CD4^+^ T sample group (4 secondary holes), 4 tubes in CD8^+^ T sample group (4 secondary holes), 4 tubes in NK cell sample group (4 secondary holes), 1 tube in CD4^+^ T FMO group, 1 tube in CD8^+^ T FMO group, 1 tube in B cell FMO group and 1 tube in NK cell FMO group. Cell concentrations of each tube were adjusted to 5x10^6^ cells/ml before adding antibody combinations to B cell, CD4^+^ T, CD8^+^ T and NK cells to the corresponding cell tube respectively (Table S4). Tubes were incubated at 4 °C for 30 min and after adding 3 ml of PBS, centrifugation was performed at 1500 rpm for 5 min before discarding the supernatant. Then, 500 μl of MitoSOX Red working solution (5 μM) was added to the ROS single positive tube and full positive sample tubes and after incubation for 20 min, at 37 °C and in the dark, the subsequent steps were conducted in the same way as mentioned in section MM detection.

**Mitophagy detection**

The whole bone marrow cells of 2 C57 mice were taken, and the leukocytes were obtained with erythrocyte lysate. For cell counting, all cells were divided into 26 tubes, and included 1 tube as the blank group, 1 tube in CD3 (BV711), 1 tube in CD25 (APC), 1 tube in CD8 (BV650), 1 tube in CD127 (APC-Cy7), 1 tube in mitophagy, 4 tubes in B cell sample group (4 secondary holes), 4 tubes in CD4^+^ T sample group (4 secondary holes), 4 tubes in CD8^+^ T sample group (4 secondary holes), 4 tubes in NK cell sample group (4 secondary holes), 1 tube in CD4^+^ T FMO group, and 1 tube in CD8^+^ T FMO group, B cell FMO group 1 tube, NK cell FMO group 1 tube. Cell concentrations of each tube were adjusted to 5x10^6^ cells/ml before adding antibody combinations for B, CD4^+^ T, CD8^+^ T and NK cells to the corresponding cell tube respectively (Table S5). Tubes were incubated at 4 °C for 30 min and after adding 3 ml of PBS, centrifugation was performed at 1500 rpm for 5 min before discarding the supernatant. Then, 500 μl of Mitophagy Dye working solution (100 nM) was added to the Mitophagy single positive tube and full positive sample tubes and after incubation for 20 min, at 37 °C and in the dark, subsequent steps were performed in a similar way as mentioned in section MM detection.

**Table S1.** Immunophenotypes of lymphocytes.

| Cell types | Cell immunophenotypes |
| --- | --- |
| pro-B | B220^+^CD43^+^ |
| pre-B | B220^+^CD43^-^IgM^-^ |
| immature B | B220^+^CD43^-^IgM^+^ |
| mature B | B220^++^CD43^-^IgM^+^ |
| naïve CD4+ T | CD3^+^CD4^+^CD25^-^CD62L^+^CD44^-^ |
| memory CD4+ T | CD3^+^CD4^+^CD25^-^CD62L^-^CD44^+^ |
| nT_reg_ | CD3^+^CD4^+^CD25^+^ |
| naïve CD8+ T | CD3^+^CD8^+^CD44^int/low^CD62L^+^ |
| central memory CD8+ T | CD3^+^CD8^+^CD62L^+^CD127^+^ |
| effector memory CD8+ T | CD3^+^CD8^+^CD62L^-^CD127^+^ |
| effector T | CD3^+^CD8^+^CD62L^-^CD127^-^ |
| NK | CD4^-^CD8^-^B220^-^NK1.1^+^ |

**Table S2.** Antibody combination scheme of mitochondrial mass detection (MitoTracker Green).

| Name of antibody combination | Antibodies |
| --- | --- |
| B | APC-Cy7 anti B220, APC anti CD43, PE-Cy7 anti IgM |
| T | PE anti CD3, PE-Cy7 anti CD4, APC anti CD25，BV510 anti CD62L, BV605 anti CD44, BV650 anti CD8, APC-Cy7 anti CD127 |
| NK | BV650 anti CD8, BV605 anti CD4, APC-Cy7 anti B220, PE-Cy7 anti NK1.1 |

**Table S3.** Antibody combination scheme of mitochondrial membrane potential detection (MitoTracker Red).

| Name of antibody combination | Antibodies |
| --- | --- |
| B | APC-Cy7 anti B220, APC anti CD43, PE-Cy7 anti IgM |
| T | BV711 anti CD3, PE-Cy7 anti CD4, APC anti CD25，BV510 anti CD62L, BV605 anti CD44, BV650 anti CD8, APC-Cy7 anti CD127 |
| NK | BV650 anti CD8, BV605 anti CD4, APC-Cy7 anti B220, PE-Cy7 anti NK1.1 |

**Table S4.** Antibody combination scheme of mitochondrial reactive oxygen species detection (MitoSOX Red).

| Name of antibody combination | Antibodies |
| --- | --- |
| B | APC-Cy7 anti B220, APC anti CD43, PE-Cy7 anti IgM |
| CD4^+^ T | BV711 anti CD3, PE-Cy7 anti CD4, APC anti CD25，BV510 anti CD62L, BV605 anti CD44 |
| CD8^+^ T | BV711 anti CD3, BV650 anti CD8, BV510 anti CD62L, APC-Cy7 anti CD127, BV605 anti CD44 |
| NK | BV650 anti CD8, BV605 anti CD4, APC-Cy7 anti B220, PE-Cy7 anti NK1.1 |

**Table S5.** Antibody combination scheme of mitophagy detection (Mitophagy Dye).

| Name of antibody combination | Antibodies |
| --- | --- |
| B | APC-Cy7 anti B220, APC anti CD43, PE-Cy7 anti IgM |
| CD4^+^ T | BV711 anti CD3, PE-Cy7 anti CD4, APC anti CD25, BV510 anti CD62L, BV605 anti CD44 |
| CD8^+^ T | BV711 anti CD3, BV650 anti CD8, BV510 anti CD62L, APC-Cy7 anti CD127, BV605 anti CD44 |
| NK | BV650 anti CD8, BV605 anti CD4, APC-Cy7 anti B220, PE-Cy7 anti NK1.1 |

**Table S6.** Information about the antibodies.

| Antibody | Clone number | Fluorochrome | Catalog No. | Company |
| --- | --- | --- | --- | --- |
| CD3 | 145-2C11 | PE | 12-0031-83 | eBioscience |
| CD3 | 17A2 | BV711 | 100241 | Biolegend |
| CD4 | GK1.5 | PE-Cy7 | 25-0041-81 | eBioscience |
| CD4 | RM4-5 | BV605 | 563151 | BD |
| CD4 | GK1.5 | APC-eFluor780 | 47-0041-82 | eBioscience |
| CD8 | 53-6.7 | APC | 17-0081-82 | eBioscience |
| CD8 | 63-6.7 | BV650 | 563234 | BD |
| CD25 | PC61.5 | APC | 17-0251-82 | eBioscience |
| CD62L | MEL-14 | BV510 | 563117 | BD |
| CD44 | IM7 | BV605 | 563058 | BD |
| CD127 | A7R34 | APC-Cy7 | 135040 | Biolegend |
| B220 | RA3-6B2 | APC-Cy7 | 103224 | Biolegend |
| B220 | RA3-6B2 | PercP-Cy5.5 | 45-0452-82 | eBioscience |
| CD43 | S7 | APC | 560663 | BD |
| IgM | II/41 | PE-Cy7 | 25-5790-82 | eBioscience |
| NK1.1 | PK136 | PE-Cy7 | 25-5941-82 | eBioscience |
